# Physicians’ attitude towards selection of second line therapy with nilotinib and dasatinib in chronic myeloid leukemia patients

**DOI:** 10.1186/s12955-017-0788-4

**Published:** 2017-10-18

**Authors:** Massimo Breccia, Michele Baccarani, Gianantonio Rosti, Francesco Cottone, Laura Cannella, François Guilhot, Marco Vignetti, Fabio Efficace

**Affiliations:** 1 Department of Biotechnologies and Hematology, University of Rome Sapienza, Rome, Italy; 2 Department of Hematology-Oncology, University of Bologna, S. Orsola-Malpighi Hospital, Bologna, Italy; 3 Italian Group for Adult Hematologic Diseases (GIMEMA), Data Center and Health Outcomes Research Unit, Rome, Italy; 4 Inserm CIC 1402 , CHU de Poitiers, Poitiers, France

**Keywords:** Chronic myeloid leukemia, Line of therapy, Tyrosine Kinase inhibitors

## Abstract

We investigated factors that physicians consider of most importance in the selection of second line tyrosine kinase inhibitors treatments (TKIs) in chronic myeloid leukemia patients (CML).

**Electronic supplementary material:**

The online version of this article (10.1186/s12955-017-0788-4) contains supplementary material, which is available to authorized users.

## Letter to the editor

The use of tyrosine kinase inhibitors (TKIs) dramatically improved the long-term outcomes of patients diagnosed with chronic myeloid leukemia (CML). Imatinib (IMA), the first example of targeted therapy, increased the overall survival up to 85%, both in controlled trial [1] and in real-life settings [2–5]. Second-generation TKIs, dasatinib (DASA) and nilotinib (NILO), were introduced in the armamentarium of active drugs in CML since 2004 after large evidence-based data supported their efficiency for rescuing IMA-resistance or IMA-intolerance in CML patients [6]. Thus far, no optimal sequencing strategy of TKI treatment in CML has been proposed due to the lack of specific comparative clinical trials [7]. Despite several factors, such as drug safety profile, comorbidities or impact on patient’s quality of life [8, 9], should be considered in the selection of second-line TKI treatment, there is no consensus on the most appropriate drug to use and very little is known on how physicians make these decisions. We conducted a pilot study investigating factors that physicians consider of most importance in treatment allocation to either NILO or DASA as 2nd line treatment allocation after IMA resistance or intolerance.

Data were collected as part of a previously published survey research on CML patients [10]. Analysis is based on a sample of 67 CML patients who switched from IMA to either NILO (*N* = 36; 53.7%) or DASA (*N* = 31; 46.3%). The protocol specified that patients had to be in second line treatment for at least 3 months to be eligible for this analysis. In all participating centers, both second-generation drugs should have been equally available for use. However, their cost was different, DASA being slightly more expensive. Fifteen physicians were involved in the management of these patients and they were asked to complete an ad-hoc eight-item questionnaire investigating reasons based on which they made the decision to either use one drug over another.

The majority of physicians interviewed were male (80%) and their median age was 44 years (range 32–62). Participating physicians had a median of 11 years (range 4–32) of experience in treating CML and of 15 years (range 5–35) of overall clinical practice. Twenty-seven percent of them reported to typically meet per week a number of CML patient between 10 to 20. (Additional file 1: Table S1).

Patient median age at the time of treatment switch was 47 (range 22–82 years) and 55 years (range 33–78) in the NILO and DASA group, respectively (*p* = 0.035). Median duration of IMA therapy for the overall population, before receiving second line therapy, was 2.3 years. Median time from treatment change to study survey was 2.75 years. No differences in the main reason for switching from IMA therapy (intolerance or resistance) or in the occurrence of any grade 3 or 4 AEs during previous IMA therapy were found between the groups of patients treated with NILO and with DASA. Physicians’ evaluation on factors that guided their decisions to switch patients to one of the two drugs was not different by type of second line therapy actually chosen (i.e., either NILO or DASA). The most relevant determinant for 2nd line TKI selection was ‘previous discussion with patients on pros and cons of drugs’, being reported as “quite a bit” or “very much” important in 73% of all evaluations. Patient’s comorbidity or personality profile, was quoted as a ‘quite a bit’ or ‘very much’ relevant reason for the selection of 2nd line TKI, respectively in 43% and 48% of all questionnaires. In spite of different prices, the cost of the drug was not considered relevant at all, in the selection of which drug to use, in 93% of the 67 evaluations considered, but it should not be overlooked that both TKIs, for many of these patients, were provided free-of-charge by the national health system. Low relevance was assigned to patient age, but patients switched to NILO were older than those switched to DASA, and to different treatment schedule: ‘not at all’ or ‘a little’ relevance was reported respectively in 64% and 70% of all questionnaires (Table 1). No statistically significant differences were found in selected factors, driving the decision to switch either to NILO or DASA when physicians considered switching from first line IMA therapy.

**Table 1:** Physician-reported reasons for choosing the 2nd line drug

Variable	Nilotinib	Dasatinib	Totaln (%)	*p*-value(2 sided)
*Accessibility of the drug in physician institution*				
Not at all	34 (94.44)	29 (93.55)	63 (94.03)	1
Very much	2 (5.56)	2 (6.45)	4 (5.97)	
*Cost of drug*				
Not at all	33 (91.67)	29 (93.55)	62 (92.54)	0.081
A little	3 (8.33)	0 (0)	3 (4.48)	
Quite a bit	0 (0)	2 (6.45)	2 (2.99)	
*Patient comorbidity profile*				
Not at all	15 (41.67)	13 (41.94)	28 (41.79)	0.977
A little	6 (16.67)	4 (12.9)	10 (14.93)	
Quite a bit	13 (36.11)	13 (41.94)	26 (38.81)	
Very much	2 (5.56)	1 (3.23)	3 (4.48)	
*Patient’s age*				
Not at all	18 (50)	13 (41.94)	31 (46.27)	0.797
A little	5 (13.89)	7 (22.58)	12 (17.91)	
Quite a bit	11 (30.56)	10 (32.26)	21 (31.34)	
Very much	2 (5.56)	1 (3.23)	3 (4.48)	
*Patient’s personality profile*				
Not at all	9 (25)	11 (35.48)	20 (29.85)	0.534
A little	7 (19.44)	8 (25.81)	15 (22.39)	
Quite a bit	18 (50)	10 (32.26)	28 (41.79)	
Very much	2 (5.56)	2 (6.45)	4 (5.97)	
*Previous discussion about Pros and Cons of both treatments*				
Not at all	5 (13.89)	3 (9.68)	8 (11.94)	0.455
A little	3 (8.33)	7 (22.58)	10 (14.93)	
Quite a bit	24 (66.67)	18 (58.06)	42 (62.69)	
Very much	4 (11.11)	3 (9.68)	7 (10.45)	
*Different treatment schedules*				
Not at all	21 (58.33)	13 (41.94)	34 (50.75)	0.615
A little	6 (16.67)	7 (22.58)	13 (19.4)	
Quite a bit	7 (19.44)	8 (25.81)	15 (22.39)	
Very much	2 (5.56)	3 (9.68)	5 (7.46)	
*Type of mutation during IM therapy*				
Not at all	31 (86.11)	26 (83.87)	57 (85.07)	0.460
Quite a bit	4 (11.11)	2 (6.45)	6 (8.96)	
Very much	1 (2.78)	3 (9.68)	4 (5.97)	

The results of this pilot survey on potential determinants for the second line TKI choice in CML showed that physicians consider discussion with their patients about advantages and disadvantages of drugs, as the most relevant factor based on which making a decision. Since the choice of the physicians is usually guided by technical and professional considerations, this suggests that the available data on efficacy, safety, and cost, were not sufficient, or were not sufficiently different, to allow a priority of physicians’ over patients’ choice. There is paucity of evidence on how such decisions are made in routine CML care and our study underscores the need of further research in this area, including prospective randomized comparative studies.

## Additional file

Additional file 1: Table S1. Physician characteristics. (DOCX 15 kb)

## Abbreviations

CML: Chronic myeloid leukemia
DASA: Dasatinib
IMA: Imatinib
NILO: Nilotinib
TKI: Tyrosine kinase inhibitors
