# Supplementary material for: Physicians’ attitude towards selection of second line therapy with nilotinib and dasatinib in chronic myeloid leukemia patients
Source: Health Qual Life Outcomes. 2017 Oct 18;15:204. doi: 10.1186/s12955-017-0788-4 (PMC5648464; doi:10.1186/s12955-017-0788-4)
Supplement: Additional file 1: Table S1. — Physician characteristics. (DOCX 15 kb) [file 12955_2017_788_MOESM1_ESM.docx]

| **Variable** | **Total (n=15)** |
| --- | --- |
| *Gender N (%)* |  |
| Male | 12 (80) |
| Female | 3 (20) |
| *Age* |  |
| Mean (SD) | 45.87 (9.79) |
| median | 44 |
| range | 32.00 - 62.00 |
| *CML patients typically visited per week N (%)* |  |
| <10 | 6 (40) |
| 10-20 | 4 (26.67) |
| >20 | 3 (20) |
| Missing | 2 (13.33) |
| *Main source of information regarding CML N (%)* |  |
| International scientific literature | 8 (53.33) |
| Conferences | 7 (46.67) |
| *Years in practice* |  |
| Mean (SD) | 18.27 (10.14) |
| median | 15 |
| range | 5.00 - 35.00 |
| *Experience in treating CML patients (years)* |  |
| Mean (SD) | 14.20 (8.07) |
| median | 11 |
| range | 4.00 - 32.00 |

**Supplemental Table 1. Physician characteristics**

*Legend:* SD=Standard Deviation
